# The Surgical Diseases of the Sub-Maxillary Gland

**Published:** 1896-07-18

**Authors:** C. Percy Paton

**Affiliations:** Surgical Registrar to Westminster Hospital


### THE SURGICAL DISEASES OF THE SUB-MAXILLARY GLAND.

By C. Percy Paton, M.D., M.S., F.R.C.S., Surgical Registrar to Westminster Hospital.

The diseases amenable to surgical interference with which the sub-maxillary glands may be affected are most of them, if not all, of somewhat rare occurrence. They may be conveniently classified for descriptive purposes into those affecting the ducts (Wharton's ducts) and those involving the gland itself.

The principal feature of the troubles affecting the *ducts* depends on the obstruction to the passage of saliva which results, and subsequently to this the inflammation which may ensue. The main affections are as follows: *Foreign bodies* occasionally find their way into the duct. These bodies are usually such elongated somewhat pointed bodies as an awn of barley or husk of oats (eaten in porridge), &c. The symptoms caused are those of irritation in the floor of the mouth, followed, if the irritant be not removed, by some dilatation of the duct and some inflammation, which may go on to suppuration, when the foreign body escapes by ulcerating through, or by the abscess being opened, after which the trouble usually subsides. Occasionally part of the offending substance can be seen before inflammation has set in protruding from the duct's orifice and so removed; but more often the inflammatory trouble is treated (*e.g.*, an abscess by incision) and the foreign body found accidentally afterwards. There may, however, have been some suspicion of its presence, if there were at the same time swelling under the jaw of the gland itself increased after taking food then subsiding again, and so distinguished from secondary inflammatory swelling of the sub-maxillary lymphatic glands. The treatment is to remove the foreign body if it can be seen, otherwise to treat the inflammatory symptoms, if possible excluding the existence of a foreign body by examining the duct through its opening with a probe. Very similar are the symptoms of a calcareous mass found *in situ*, viz., a *salivary calculus*. Various causes have been assigned to its formation, as a foreign body, tartar from the teeth, retention of saliva from any cause, &c. The stone is almost always single, and composed of phosphates and carbonates of lime, with the addition sometimes of small quantities of urates. Some extraneous body may form its nucleus. The symptoms to which it gives rise are various. Sometimes during its formation there are none, and it may be found accidentally on the floor of the mouth by the patient, who wonders where it came from. More usually, however, there is a good deal of pain and swelling in the floor of the mouth, with, perhaps, some dryness. At the same time the gland itself becomes enlarged, and there is an acute enlargement whenever its secretory activity is stimulated by taking food. Occasionally, if more active inflammation is caused, this may spread to the cellular tissue of the upper part of the neck, causing a sort of phlegmon. This, however, is rare. Periodical attacks of spasmodic pain have also been described as salivary colic, and have been thought, though perhaps with doubtful reason, to be analogous to renal or biliary colic.

The diagnosis of stone is usually not very difficult, as palpation, carefully carried out, will usually reveal the stone, and passing a fine probe along the duct will settle the question by striking against it. Doubt will usually only arise when there is much inflammation, in which case it may be confused with periostitis of the jaw, or even tumour; but here an incision will usually soon set any doubts at rest.

The treatment of calculus is, of course, to remove the stone, and the greatest pains should be taken to do so, if possible, without breaking it, as a few small pieces left often cause more irritation than the whole of the smooth complete calculus. This may sometimes be done by dilating the orifice of the duct, but there need be no hesitation in incising the mucous membrane over it if necessary, and, indeed, this is almost certain to be necessary if there has been any inflammatory trouble.

Two other affections of Wharton's duct may be mentioned. The first is catarrhal inflammation. This has been said to spread from the mouth and cause swelling of the gland from obstruction of the duct at meal times. If it occurs, it is very rare, and is of very little clinical importance apart from the condition of the mouth itself.

The other affection is the more common one of Ranula; this, however, is only very occasionally due to dilatation of the sub-maxillary duct, being much more often due to swelling of the Blandin-Nuhn or other mucous gland in the neighbourhood. When present it forms a bluish, obviously fluid swelling on one or other side of the frænum linguæ. It is often rather obstinate to treatment, which can only just be indicated here, two very useful methods being the use of a seton, and cutting out a considerable piece of the wall of the cyst, and then stitching a triangular corner of it down into the depths of the cavity, so as to compel it to drain into the mouth.

The affections of the gland itself are few in number. The inflammatory troubles include the following: *Acute inflammation* may occur in the gland of a phlegmonous or suppurative nature. This may be secondary to an inflammation in the surrounding neighbourhood, or it may be pyæmic in origin. In either case it is not frequently seen, and is best treated on the general principles which would guide one in dealing with a similar condition elsewhere. *Tuberculous* and *gummatous* inflammations have also been described as attacking the gland. Mumps also attacks the submaxillary, but is of no special surgical importance.

Several varieties of *new growth* have been found growing in connection with the gland. Adenomata, euchondromata, sarcomata, carcinomata, and mixed tumours have all been found in this situation; the most common, however, is the last-named, which is similar to that found in the parotid, and consists of myxomatous tissue, along with cartilage and glandular tissue. It is usually non-malignant, slow in growth, and often contains cysts in its substance, which are usually small in size; sometimes, however, it takes on malignant growth. The diagnosis of sub-maxillary new growth is by no means easy, as enlargement of lymphatic glands in this neighbourhood is so very much more common. The consistency of most sub-maxillary tumours is, however, very much harder than that of lymphatic swellings, which latter show a great tendency to break down if they are tuberculous. This, taken with the exact situation of the swelling, its nearness to the mucous membrane when examined through the floor of the mouth, and the absence of similar swellings in other parts of the neck where lymphatic glands are abundant, will usually enable a diagnosis to be arrived at.

A few anatomical points must now be referred to which should be kept in mind on removing these tumours. In the first place, it must be remembered that the gland consists of two parts—one which lies merely beneath the platysma and deep fascia, and a deeper portion which is beneath the mylo-hyoid muscle and close to the floor of the mouth. The former part will be at once exposed by raising a semicircular flap consisting of the structures down to and including the deep fasein, but to expose the deeper part the mylo-hyoid must be divided. This deeper part may not be involved in the growth, in which case, if the tumour be simple, it can be left. The facial artery also must not be forgotten as curving around and partially through the more superficial part of the gland, and will necessarily be divided in its removal. The gland lies also upon the lingual and hypoglossal nerves, while just posterior to it are the great vessels of the neck. The cavity of the pharynx, too, in its deep aspect, is quite close at hand. None of these structures will, however, be in any serious danger in simple tumours, which easily shell out, but in malignant growths they may all be involved in the spread of the new growth, which in malignant cases, after having escaped from the gland capsule, involves all kinds of structures in its spread indiscriminately.

